# Polyphenol Profile and Biological Activity of the Extracts from *Sideritis scardica* Griseb. (Lamiaceae) Herb

**DOI:** 10.3390/ph18081121

**Published:** 2025-07-27

**Authors:** Magdalena Walasek-Janusz, Krzysztof Kamil Wojtanowski, Rafał Papliński, Agnieszka Grzegorczyk, Renata Nurzyńska-Wierdak

**Affiliations:** 1 Department of Vegetable and Herb Crops, Faculty of Horticulture and Landscape Architecture, University of Life Sciences in Lublin, 50A Doświadczalna Street, 20-280 Lublin, Poland; magdalena.walasek@up.lublin.pl (M.W.-J.); rafal.paplinski@up.lublin.pl (R.P.); 2 Department of Pharmacognosy and Pharmaceutical Botany, Medical University of Lublin, 1 Chodzki Street, 20-093 Lublin, Poland; krzysztof.wojtanowski@umlub.pl; 3 Department of Pharmaceutical Microbiology, Faculty of Pharmacy, Medical University of Lublin, 1 Chodzki Street, 20-093 Lublin, Poland; agnieszka.grzegorczyk@umlub.pl

**Keywords:** herbal extracts, variability of chemical composition, antioxidant activity, antimicrobial activity

## Abstract

**Background/Objectives:** The beneficial and multifaceted effects of *Sideritis scardica* Griseb. extracts are attributed to the presence of polyphenolic compounds, particularly phenolic acids. **Methods:** The research was carried out for *S. scardica* herb of different origins (Albania, Bulgaria, North Macedonia, and Türkiye). Identification of compounds was performed using the HPLC/ESI-QTOF-MS method; phenolic acids and flavonoids were determined spectrophotometrically. The antioxidant activity of methanol extracts from studied herbs was determined using the Folin–Ciocalteu, DPPH, and FRAP methods, and the antimicrobial activity was evaluated using the broth microdilution method in accordance with the guidelines of the European Committee on Antimicrobial Susceptibility Testing (EUCAST). **Results:** We demonstrated the presence 18–20 active compounds, depending on the origin of the raw material, with verbascoside being the predominant compound in all samples. The raw material was characterized by significant polyphenol content and high antioxidant activity. DPPH tests revealed the highest antioxidant activity, ranging from 86.5% to 87.9%, in samples from Bulgaria, North Macedonia, and Türkiye, and the latter showed the strongest antimicrobial activity, particularly against Gram-positive pathogens and Candida spp. **Conclusions:** This research is the first report comparing the chemical composition and biological activity of *S. scardica* raw material of different origins. Our findings indicate that *S. scardica* herb extracts have significant phytotherapeutic potential, although this varies depending on the origin of the raw material, and point to the need for further research on this plant material, particularly in terms of the level of active compounds and their possible synergistic effects with conventional drugs, as well as the need for standardization.

## 1. Introduction

The genus Sideritis (Lamiaceae) includes aromatic, annual and perennial herbaceous plants, and small shrubs diversified in terms of morphology and chemistry, growing at different altitudes and in different habitats. Wild populations of Sideritis develop on sunny, steep slopes and pastures, from a few to over 3000 m above sea level [1,2,3]. In recent years, in-depth studies have focused on the Sideritis genus, particularly regarding its botanical, phytochemical, and pharmacological characteristics. All of its species have been found to contain significant amounts of terpenes, phenolic compounds, and essential oils, i.e., chemical constituents responsible for their multifaceted pharmacological activity [2,3]. Due to its unique medicinal properties, the interest in *Sideritis* spp. herbs is constantly growing, both in the European and global markets. One of the representatives of the Sideritis genus is *Sideritis scardica* Griseb. (synonyms: *S. florida* (Boiss. & Heldr.), *S. raeseri* subsp. *florida* (Boiss. & Heldr.; Papan. & Kokkini)) [4], endemic to the Balkan Peninsula, known in the southeastern part of Europe for traditional uses: alleviating colds, strengthening the body, and as a sedative. The species occurs in rocky areas and in the alpine regions of northern Greece up to the Olympus mountain range and Pelion [4,5,6]. It is a perennial plant, with a simple or branched stem, lanceolate leaves covered with white tomentose, with yellow, bell-shaped, densely haired flowers [3]. The above-ground parts of sideritis (referred to as sideritis herb) are collected mainly from the wild during flowering. They are very popular in Mediterranean countries, widely available in shops and at local markets. Known as “mountain tea” or “shepherd′s tea,” they have been used in traditional medicine since ancient times for their antimicrobial, analgesic, antispasmodic, carminative, and antidiabetic effects [3,7]. Today, infusions and decoctions of the Sideritis herb are prepared for medicinal purposes and used internally to aid digestion; strengthen the immune system; treat flu, sinusitis, allergies, pain, and anxiety; as well as locally in the form of compresses or therapeutic baths [2].

*Sideritis* species are considered promising therapeutic agents in the treatment of upper respiratory tract and peptic ulcer/inflammatory diseases [8]. The European Medicines Agency (EMA) has recognized *Sideritis* spp. as medicinal plants within the framework of traditional use. Infusions of *S. scardiaca* Griseb., *S. clandestina* (Bory & Chaub.) Hayek, *S. raeseri* Boiss. & Heldr., and *S. syriaca* L. are acknowledged for their use in the treatment of colds, relieving coughs, and mild gastrointestinal disorders [1,9]. Extracts of *S. scardica* represent a rich source of polyphenols and exhibit pronounced antioxidative properties [10]. The total polyphenol content in sideritis herb reaches 240 g per kg of dry weight, contributing to its multiple health benefits [3]. The broad-spectrum biological effects of *S. scardica* extracts are believed to result from the presence of polyphenolic compounds, particularly phenolic acids [11,12,13]. In a study conducted by Behrendt et al. [5], supplementation with *S. scardica* extract combined with B-group vitamins over a six-week period was shown to reduce the effects of mental stress and improve stress resilience, cognitive efficiency, and visual attention under acute stress conditions. According to the authors, these findings may be of particular relevance to individuals performing cognitive tasks in high-conflict or noisy environments (e.g., open-plan offices or while driving). The pharmacological profile of *S. scardica* extracts suggests potential applications in phytotherapeutic approaches for mental health conditions such as anxiety disorders, major depression, attention deficit hyperactivity disorder (ADHD), intellectual disability, or neurodegenerative diseases [14,15]. Furthermore, certain compounds isolated from Sideritis spp. have demonstrated antiproliferative, cytotoxic, and anti-HIV activities [1,16].

Extracts from sideritis herb contain three main classes of active compounds: essential oils (0.02–0.83%), diterpenes (0.33%), and polyphenols (3.19–12.38 mg GAE·g^−1^ DM) [17]. Mróz et al. [18] conducted a comparative analysis of the phytochemical profiles of various extracts of *S. raeseri* and *S. scardica* and classified the major groups of identified secondary metabolites as flavonoids, terpenoids, phenylethanoid glycosides, and phenolic acids. The principal antioxidants in these extracts were derivatives of isoscutellarein and hypolaetin, as well as verbascoside and chlorogenic acid. Among the tested solvents, 70% ethanol proved to be the most effective extractant for various classes of phytochemicals, including antioxidants. Chemical characterization of n-hexane extracts obtained from the aerial flowering parts of *S. scardica* (Macedonia) and *S. raeseri* (Macedonia and Albania) revealed the presence of over 90 components, predominantly diterpenes and hydrocarbons. The most abundant components were hentriacontane, nonacosane, heptacosane, and two unidentified compounds, presumed to be diterpenes [19]. The total polyphenol content and antioxidant activity, as determined using the DPPH method, were 50.8 mg GA/g and 3.2–8.9 mg/mL for *S. scardica*, and 48.9 mg GA/g and 7.6–12.6 mg/mL for *S. raeseri*, respectively. The high cross-pollination capacity within the genus Sideritis contributes to the emergence of numerous phenotypes. A phenotypic assessment of *S. scardica* populations from six sites located in northern Greece revealed considerable morphological variability in leaf and inflorescence traits, with a coefficient of variation higher than 15% [4]. High phenotypic variability in sideritis plants may also be related to chemical variability, and this consequently causes fluctuations in the quality of herbal products. Sarrou et al. [20] showed a comparable polyphenolic profile across sideritis extracts, with chlorogenic acid and verbascoside being the dominant constituents, at concentrations ranging from 134.88 to 230.00 and from 1511.51 to 2234.32 mg/100 g, respectively. Similarly, Stanoeva et al. [21] investigated the polyphenolic profile, including the phenolic acids, phenylethanoid glycosides, and flavonoids, in 42 samples representing four Sideritis species from various regions of the Balkan Peninsula in order to reveal correlations between their taxonomy, geographical location, and the profile and content of polyphenolic compounds. They demonstrated that the differences observed in the phenolic profile depended more on the geographical location and climate than on the species. The aim of the present study was to evaluate the phytotherapeutic potential of *S. scardica* extracts derived from commercial raw materials originating from Albania, Bulgaria, Macedonia, and Türkiye. The evaluation was based on the analysis of their phytochemical profiles using high-performance liquid chromatography coupled with electrospray ionization quadrupole time-of-flight mass spectrometry (HPLC/ESI-QTOF-MS), as well as the quantification of biologically active compounds—including total polyphenols, flavonoids, and phenolic acids—and the assessment of their antioxidant and antimicrobial activities. To the best of our knowledge, this is the first study to evaluate the phytotherapeutic value of sideritis raw materials available through official retail channels (herbal/medical stores), taking into account their chemical composition and biological activity in the context of potential geographical origins.

## 2. Results

### 2.1. HPLC-DAD-MS/MS Analysis of the S. scardica Herb

**Figure 1:**
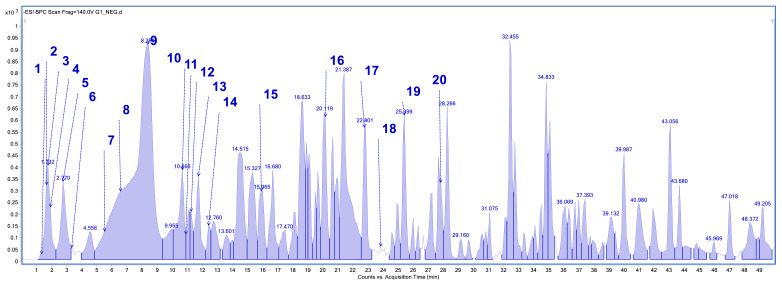
Base peak chromatogram of the *S. scardica* extract (Sh1; Bulgaria) by high-performance liquid chromatography–electrospray ionization quadrupole time-of-flight mass spectrometry (HPLC/ESI-QTOF-MS). Identified compounds (1–20) are summarized in Table 1.

**Figure 2:**
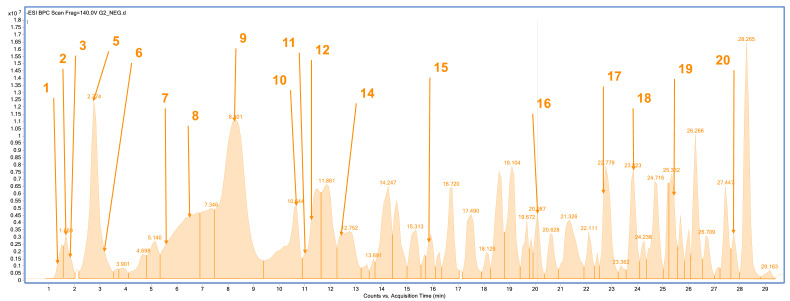
Base peak chromatogram of the *S. scardica* extract (Sh2; Türkiye) by high-performance liquid chromatography–electrospray ionization quadrupole time-of-flight mass spectrometry (HPLC/ESI-QTOF-MS). Identified compounds (1–20) are summarized in Table 1.

**Figure 3:**
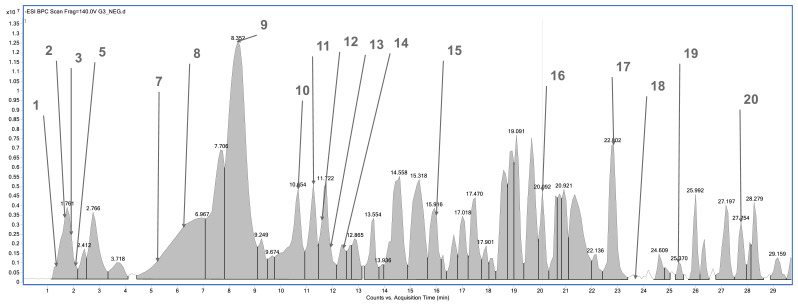
Base peak chromatogram of the *S. scardica* extract (Sh3; Macedonia) by high-performance liquid chromatography–electrospray ionization quadrupole time-of-flight mass spectrometry (HPLC/ESI-QTOF-MS). Identified compounds (1–20) are summarized in Table 1.

**Figure 4:**
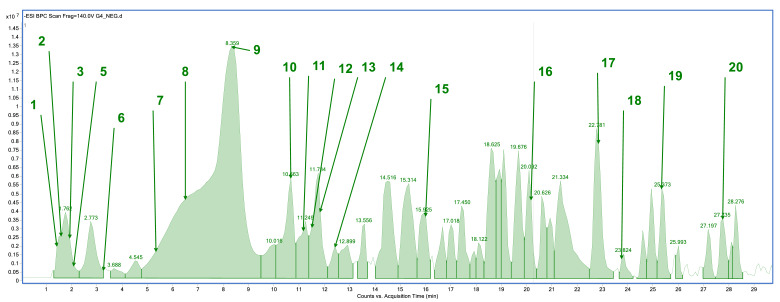
Base peak chromatogram of the *S. scardica* extract (Sh4; Albania) by high-performance liquid chromatography–electrospray ionization quadrupole time-of-flight mass spectrometry (HPLC/ESI-QTOF-MS). Identified compounds (1–20) are summarized in Table 1.

The plant material was subjected to an extraction process using methanol as a solvent. Phytochemical profile analysis performed using high-performance liquid chromatography coupled with electrospray ionization quadrupole time-of-flight mass spectrometry (HPLC/ESI-QTOF-MS) revealed the presence of 18 to 20 compounds in the analyzed extracts (Figure 1, Figure 2, Figure 3 and Figure 4). The extract obtained from the aerial parts of sideritis (Sh1; from Bulgaria) contained the highest number of identified compounds (20; Figure 1, Table 1), with verbascoside being the predominant constituent. Notably, the extract from Bulgarian sideritis (Sh1) was the only sample to contain citric acid. Extracts Sh2 (Türkiye) and Sh3 (Macedonia) each contained 18 compounds, whereas 19 compounds were identified in extract Sh4 (Figure 4). Across all analyzed samples, phenylethanoid glycosides, particularly verbascoside, were the major phytochemicals detected. In addition to verbascoside derivatives such as isoacetoside and alyssonoside, the Sh extracts also contained various flavonoids (e.g., apigetrin) and organic acids, including malic acid and chlorogenic acid. Furthermore, extract Sh2 exhibited a comparatively higher concentration of chlorogenic acid than the other analyzed extracts.

### 2.2. Biological Activity of the Extract Obtained from Sideritis Herb

#### 2.2.1. Polyphenol Profile and Antioxidant Activity

**Figure 5:**
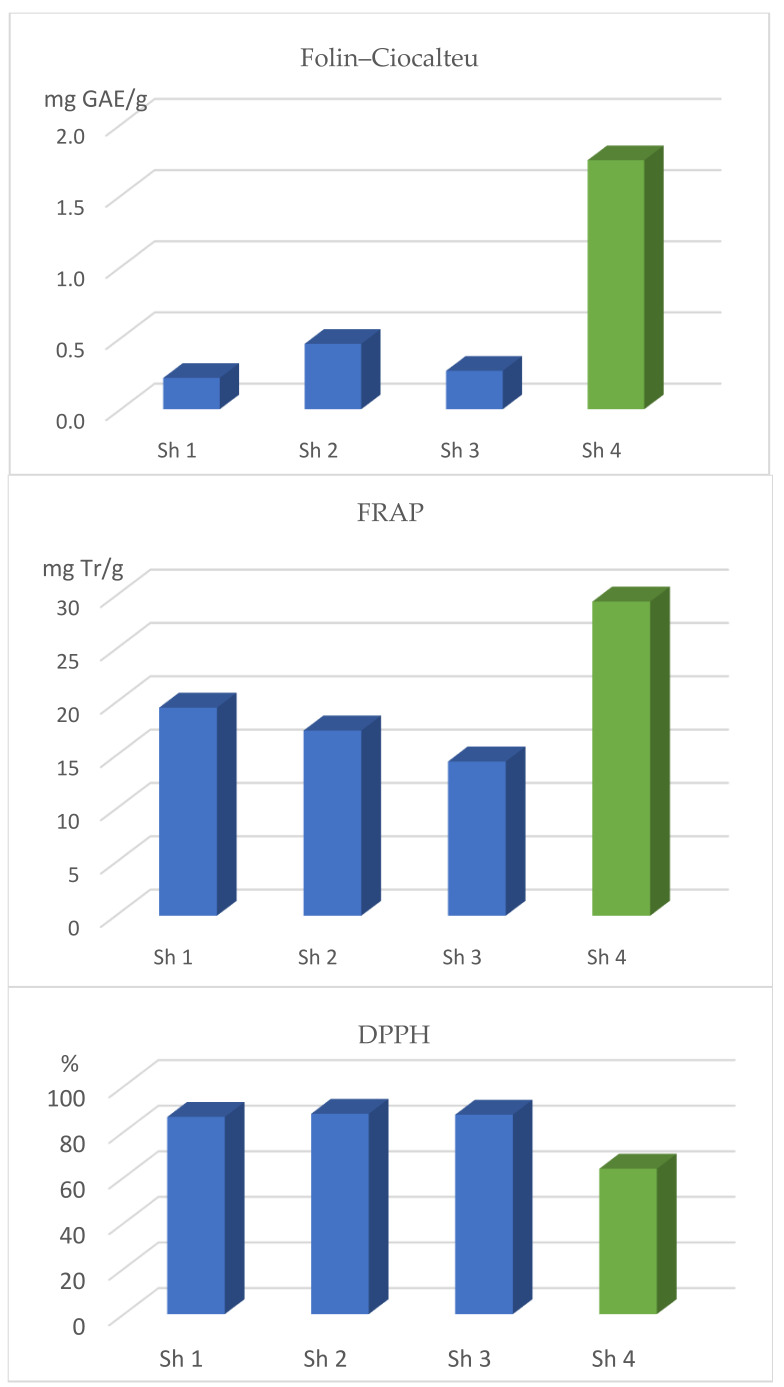
The antioxidant activity of the studied sideritis herb extracts was evaluated using multiple analytical methods (Sh1—herb from Bulgaria; Sh2—herb from Türkiye; Sh3—herb from Macedonia; Sh4—herb from Albania). Bars of the same color represent values that did not differ significantly.

The examined Sideritis herb was distinguished by the significant presence of polyphenols and antioxidant activity, which varied depending on the origin of the raw material (Table 2; Figure 5). The Albanian Sideritis herb was characterized by the highest concentration of flavonoids converted to quercetin (6.18 mg/g). The content of phenolic acids expressed as caffeic acid ranged from 11.4 to 21.8 mg/g and was not significantly different in the Albanian, Macedonian, and Türkiye raw material. The antioxidant activity of Sideritis herb extracts was as follows: according to the Folin–Ciocalteu and FRAP tests, it was the highest in the extracts from the Albanian raw material (respectively: 1.75 mg GAE/g and 29.5 mg Tr/g), whereas in the evaluation according to the DPPH test, the highest activity (86.5–87.9%) was found for the Bulgarian, Macedonian, and Türkiye raw materials.

#### 2.2.2. The Antimicrobial Activity of the Sideritis Herb Extract

**Figure 6:**
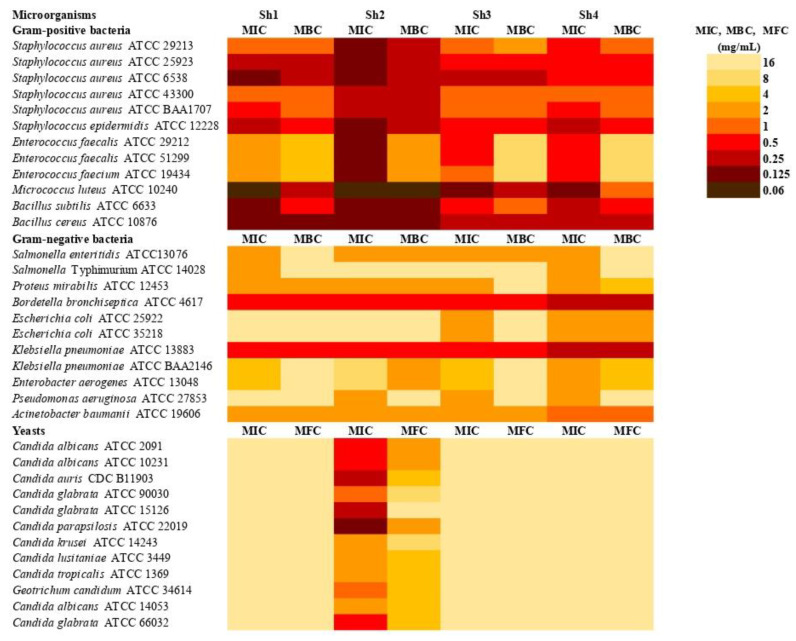
Antimicrobial activity (MIC doses) of *S. scardica* extracts against tested microorganisms (Sh1—herb from Bulgaria; Sh2—herb from Türkiye; Sh3—herb from Macedonia; Sh4—herb from Albania).

The present study evaluated the antimicrobial potential of four extracts from *S. scardica* (designated Sh1–Sh4) against a wide spectrum of clinically relevant microorganisms, including Gram-positive and Gram-negative bacteria, as well as fungal pathogens. The MIC, MBC, and MFC values presented in the heatmap (Figure 6) provide a comprehensive profile of the differential activity exhibited by these extracts.

Among Gram-positive bacteria, extract Sh2 exhibited the most pronounced activity, particularly against *Micrococcus luteus* (MIC = 0.06 mg/mL), *Staphylococcus aureus* ATCC: 29213, 25923, 6538, *S. epidermidis*, and Bacillus spp. (MIC = 0.125 mg/mL), with corresponding MBC/MIC ratios ≤ 4, confirming bactericidal properties. Interestingly, even the methicillin-resistant S. aureus (MRSA; ATCC 43300, BAA1707) showed susceptibility to Sh2 (MIC = 0.25 mg/mL), again with a bactericidal effect. A more moderate effect of Sh2 was observed against Enterococcus spp. (MIC = 0.125 mg/mL), although the MBC/MIC ratios > 4 indicated a bacteriostatic mode of action. In contrast, extract Sh1 exhibited bactericidal activity against *Enterococcus faecalis* ATCC 29212 (MIC = MBC = 0.125 mg/mL), suggesting extract-specific variation in bioactivity.

All extracts demonstrated bactericidal activity against Gram-negative bacteria. Sh2 again showed the highest activity, particularly against *Bordetella bronchiseptica* and *Klebsiella pneumoniae* ATCC 13883, with an MIC of 0.5 mg/mL. It is noteworthy that Sh2 retained activity against other multidrug-resistant strains such as *Acinetobacter baumannii*, although at higher MICs (2 mg/mL). These findings indicate a broad-spectrum potential for this extract, even against less permeable Gram-negative outer membranes.

In the case of antifungal activity, Sh2 showed the highest activity, especially against *Candida parapsilosis*, *C. auris*, and *C. glabrata* ATCC 15126 (MIC = 0.125–0.25 mg/mL). However, despite the low MICs, the MFC/MIC ratios exceeded 4, indicating fungistatic rather than fungicidal action. Notably, Sh2 also demonstrated moderate activity (MIC = 0.5 mg/mL) against *C. albicans* ATCC: 2091, 10231 strains, and *C. glabrata* ATCC 66032. In contrast, extracts Sh1, Sh3, and Sh4 were expressly less successful (MIC = 16 mg/mL), though their MFC/MIC ratios of 1 confirmed fungicidal properties.

## 3. Discussion

Extraction—the basic process of separating active substances from inert/inactive material—requires the use of an appropriate solvent and extraction procedure. Extraction parameters are selected depending on the type of raw material and bioactive substance. Yanchev et al. [10] used different extraction procedures for *S. scardica* and obtained the highest values of total polyphenols and total flavonoids in 70% ethanol extracts, followed by water/ethanol mixtures and aqueous decoctions. The highest antioxidant potential was found in extractions with water and 70% ethanol solution, where the amount of polyphenols was also sufficient. The results obtained in this study regarding total polyphenol content (4.0–32.2 mg GAE/g dry plant material) and flavonoid content (2.15–6.18 mg QE/g dry plant material) in the aerial parts of *S. scardica* are consistent with previous literature data, where flavonoid levels were reported in the range of 1.33–5.14 mg QE/g dry material.

In comparison, methanol extracts of *S. rubriflora* from Türkiye exhibited significantly higher flavonoid content, reaching 155.7 mg/g, while extracts from *S. congesta* and *S. vuralii* showed lower, yet still higher values than in our study (31.7 and 14.2 mg/g, respectively) [22]. Notably, among the samples analyzed in the present research, the highest flavonoid content (6.18 mg/g) was observed in the sample originating from Albania (Sh4), whereas the sample from Türkiye contained 2.5 mg/g. This value is substantially lower than those reported for other Turkish Sideritis species. Similarly, low levels of phenolic compounds were noted in tea prepared from hybrid plants (*S. scardica × S. syriaca*), where the flavonoid content was 9.6 mg/g and the total polyphenol content reached 32.2 mg/g [23]. In a study conducted by Irakli et al. [24], the total polyphenol content of *S. scardica* aerial parts was determined using an ultrasound-assisted extraction method with water infusion. The obtained value was 32.2 mg GAE/g of dry plant material, which is consistent with the results reported by Alipieva et al. [23], who found 29.20 mg GAE/g in a methanolic extract. These findings suggest that ultrasound-assisted extraction may facilitate the efficient extraction of polyphenolic compounds.

According to the literature, the total content of phenolic compound in sideritis herb can also vary significantly depending on the species [8,13,17]. In the study conducted by Sevindik et al. [22], which investigated several Sideritis species including *S. rubriflora*, *S. libanotica* subsp. *violasens*, *S. brevidens*, *S. erythrantha* var. *cedretorum*, *S. congesta*, and *S. vuralii* from Türkiye, the total phenolic content ranged from 35.5 to 366.9 mg/g. The highest values were recorded for extracts obtained from *S. erythrantha* subsp. *cedretorum* and *S. rubriflora*, reaching 366.9 and 328.3 mg/g dry weight, respectively. These are substantially higher levels compared to the present study, in which the total phenolic content in sideritis herb of Türkiye origin (sample Sh2), as determined by the Folin–Ciocalteu method, was 0.46 mg GAE/g of raw material. This was among the highest values recorded in our study, surpassed only by the sample from Albania (Sh4), which exhibited a total phenolic content of 1.75 mg GAE/g. In the study by Tadić et al. [11], the total polyphenol content in ethanol extracts of *S. scardica* Griseb. reached 188.5 mg/g, while Nakiboğlu et al. [25] reported a value of 0.089 μg GAE/μg extract for methanol extracts of *S. spylea*. Additionally, Uysal et al. [26] determined a total polyphenol content of 129.75 mg/g and a total flavonoid content of 111.47 mg/g in *S. libanotica* subsp. *kurdica* from Iraq.

A positive correlation was found between polyphenol content and antioxidant activity. In the study conducted by Uysal et al. [26], the ethanol extract of *S. libanotica* subsp. *kurdica* from Iraq (Duhok) demonstrated high antioxidant activity, with a DPPH radical scavenging effect of 75.15% ± 1.45 at a concentration of 2 mg/mL. Similarly, the methanol extracts analyzed in the present study exhibited strong DPPH radical scavenging capacity, ranging from 63.8% to 87.9%. The highest antioxidant activity, as measured by DPPH inhibition, was observed in the extracts from Türkiye and Macedonia at 87.9% and 87.5%, respectively. In contrast, Sevindik et al. [22] reported significantly lower DPPH scavenging activity for extracts of *S. congesta* and *S. brevidens*, with values of 39.1% and 38.9%, respectively.

In the case of antioxidant activity measured using the FRAP method, various Sideritis species originating from Türkiye demonstrated activity levels ranging from 970.8 to 1500.2 μmol/g [22]. Similarly, high antioxidant activity assessed by ferric ion reducing ability (FRAP), ranging from 14.5 to 29.5 mg Trolox/g, was observed in the present study. The highest activity was recorded for the sample Sh4 (Albania). These findings indicate that the content of polyphenolic compounds, flavonoids, as well as antioxidant activity in Sideritis species is highly variable and depends both on the geographical origin and the specific species studied. This also indicates the need to standardize the sideritis raw material based on the polyphenol content or specific compounds from this group.

Our research demonstrates not only the strong antioxidant and antimicrobial activity of Sideritis extracts but also the considerable variability of both the raw material and the and the resulting extracts. The differences in the chemical composition of *S. scardica* and *S. raeseri* originating from various geographical regions may be attributed to environmental factors, as well as to the natural tendency of some Sideriris species to interbreed [27]. *S. scardica* extracts and their component verbascoside have shown cognitive enhancement, stress-protective, neuroprotective, neurogenetic, anxiolytic, anti-aging, anti-inflammatory, antimicrobial, gastroprotective, glycemic, anti-obesity, antioxidant, and anticancer effects [28,29]. We confirmed that the main detected phytochemicals of *S. scardica* herb were phenylethanoid glycosides, especially verbascoside (also known as acteoside). The extract obtained from the Bulgarian raw material contained the largest number of identified compounds (20), with verbascoside being the dominant one. Moreover, this extract was the only one to contain citric acid. The extracts from Türkiye and Macedonian sideritis contained 18 compounds each, while in the extract from Albanian Sideritis, 19 compounds were identified including numerous flavonoids (e.g., apigetrin) and organic acids, including malic acid and chlorogenic acid. The sideritis extract from Türkiye showed relatively higher concentrations of chlorogenic acid compared to the others. Kaparakou et al. [30], analyzing hydroalcoholic extracts from Sideritis species (*S. raeseri*, *S. scardica*, and *S. syriaca*) originating from Greece using LC-MS/MS-QTOF analysis, identified the presence of 23 secondary metabolites, including 17 flavonoids, 4 phenylethanoid glycosides, 1 phenolic acid, and 1 fatty acid. Among the identified compounds, verbascoside (a phenylethanoid glycoside) and selected flavonoids such as apigenin 7-O-glucoside and isoscutellarein 7-O-[6″-O-acetyl]-allosyl(1→2)-glucoside were found in all samples. The presence of verbascoside as the main active compound in Sideritis herb was also confirmed in the present study, along with flavonoids as the dominant class of compounds. Bardakci et al. [31] analyzed the extracts of *S. congesta* P.H.Davis & Hub.-Mor. and demonstrated that the ethyl acetate fraction contained the highest polyphenol content, the strongest antioxidant activity, as well as the highest content of verbascoside and martinoside, before the water fraction. The R-H_2_O fraction contained seven compounds, including the phenylethanoid glycoside, verbascoside, two flavonoids, stachyspinoside, isoscutellarein 7-O-(6‴-O-acetyl)-β-allopyranosyl-(1→2)-β-glucopyranoside, the phenolic compound chlorogenic acid, the iridoid glycoside ajugoside, and a mixture of monoterpenoid glucosides: betulalbuside A and 1-hydroxylinaloyl 6-O-β-D-glucopyranoside.

Gioran et al. [28] showed that sideritis extracts exhibit not only antioxidant activity but also other bioactivities relevant to neuroprotection. Their study demonstrated that *S. clandestina* subsp. *peloponnesiaca*, although a weaker antioxidant compared to other Sideritis spp., has antiaggregation activity. The authors identified two pure compounds—sideridiol and verbascoside—as responsible for these beneficial effects. The results support the potential use of mountain tea in the elderly population for dementia treatment and demonstrate the activity of sideridiol against Aβ aggregation, which can be used for drug development. Importantly, treatment with sideridiol did not induce adverse effects in animal models, consistent with its lack of toxicity. Phenotypic analysis of the animals treated with verbascoside indicated a low but still existing toxic potential. Overall, no significant changes were observed in the measured traits (except at specific concentrations of verbascoside), which is consistent with the lack of toxicity of the extracts and compounds tested.

Sideritis extracts represent promising herbal remedies with a broad spectrum of biological potential. These extracts inhibit amyloid-β aggregation and toxicity in *Caenorhabditis elegans*, which was used as a model of Alzheimer′s disease. Midpolar extracts (40 and 50% ethanol) were the most active, reducing plaque count by 21% and delaying amyloid-β-induced paralysis by up to 3.5 h [15]. Evaluation of hexane extracts from wild *S. scardica* and the cultivated *S. scardica* × *S. syriaca* hybrid showed that the main components of all extracts were diterpenes and n-alkanes. None of them were active against Gram-positive *E. coli* and the fungus *C. albicans*, but all demonstrated good activity against *Staphylococcus aureus*. Interestingly, the antibacterial activity of wild and cultivated plants did not differ significantly. The weakest antibacterial activity was observed in the sample with the lowest diterpene concentration [32]. In this study, the Sh2 extract, which exhibited the strongest and most effective broad-spectrum activity, was particularly effective against both Gram-positive and Gram-negative bacteria, as well as against fungal strains such as *Candida auris* and *C. glabrata*. These results may indicate the high content of phenolic acids and chlorogenic acid in this extract, compounds known for their antimicrobial efficacy [10,13]. Verbascoside, the main component detected in all extracts, especially Sh1 and Sh2, is widely known for its bactericidal and antifungal activity through mechanisms such as cell membrane disruption and inhibition of bacterial enzymes. Furthermore, the presence of isoacetoside and forsythoside B—phenylethanoid glycosides structurally related to verbascoside—may enhance the observed synergistic antimicrobial activity [31]. The moderate to strong activity of the Sh2 extract against MRSA and *Acinetobacter baumannii*, which are known multidrug-resistant pathogens, suggests the potential of *S. scardica* extracts as a complementary strategy for treating resistant infections. Similar results have been previously reported for other Sideritis species, where high levels of polyphenols, particularly flavonoids and phenolic acids, correlated with antimicrobial potential [33]. Interestingly, although the Sh1 extract was less active against Gram-negative strains and fungi, it exhibited targeted bactericidal activity against *Enterococcus faecalis*, suggesting possible extract- or compound-specific interactions. This confirms previous observations that variability in the antimicrobial efficacy of Sideritis extracts may depend on geographic origin and phytochemical composition [34]. Overall, the results support the ethnopharmacological use of *S. scardica* and highlight the potential of its polyphenol-rich extracts as natural antimicrobials.

Antimicrobial and anti-inflammatory properties have been attributed to verbascoside [29]. Our research confirms these associations and further suggests a possible role for chlorogenic acid. The most active and broadest-spectrum extract, Sh2, was characterized not only by the presence of verbascoside but also by the highest chlorogenic acid content. Chlorogenic acid exhibits diverse antimicrobial effects, making it a potential preservative and food additive. This phenolic compound also exerts an inhibitory effect on multidrug efflux systems of multidrug-resistant bacteria and their biofilm formation, as well as an antifungal effect against *C. albicans* by having an impact on the fungi′s cell membrane [35].

In summary, our results indicate that the Sh2 extract (Türkiye) demonstrates the greatest therapeutic potential, particularly against Gram-positive pathogens and Candida species, acting primarily through bactericidal and fungistatic mechanisms. The observed variability in antimicrobial activity among the extracts highlights the influence of phytochemical composition, which is likely affected by factors such as solvent type, polarity, and extraction conditions. Further studies involving detailed phytochemical profiling and investigations into mechanisms of action are necessary to identify the active constituents responsible for this antimicrobial activity and to evaluate their potential synergy with conventional antibiotics or antifungal agents. Given the observed variability, future research should focus on the identification and quantification of specific bioactive compounds. Although considerable research has been conducted on *S. scardica* extracts, the precise compounds responsible for the reported biological effects remain insufficiently characterized. Among the identified candidates, sideridiol (from ethyl acetate fractions) and verbascoside (from methanol extracts) appear particularly promising [28].

In conclusion, our findings on the chemical composition and biological activity of methanolic extracts from *S. scardica* of different geographical origins provide valuable insights that may support the development of herbal medicinal products and dietary supplements.

## 4. Materials and Methods

### 4.1. Plant Material

The research material was the herb of *Sideritis scardica* Griseb. originating from Bulgaria (Sh1), Türkiye (Sh2), Macedonia (Sh3), and Albania (Sh4), which was suitably crushed and intended for further studies to determine the content of secondary metabolites (phenolic acids, flavonoids, polyphenols) and to determine their biological activity, including microbiological activity and antioxidant activity. The raw material for research was purchased in herbal-medical stores located in Lublin (Poland). Sample designations, origins of the raw material, and their producers are given in Table 3.

### 4.2. Identification of Compounds with the HPLC/ESI-QTOF-MS Method

The plant material underwent triple extraction (30 min each) using 20 g of ground *S. scardica* herb and 100 mL of methanol. The resulting extracts were filtered and subsequently evaporated using a rotary evaporator (Rotary Evaporator 05-ST; IKA-Werke GmbH & Co., Staufen, Germany). The resulting dense extracts were then used to prepare samples for compound identification via the HPLC/ESI-QTOF-MS technique. For this purpose, approximately 0.1 g of the dense extract was weighed and dissolved in 10 mL of methanol using an ultrasonic bath.

The purified samples were analyzed qualitatively by an HPLC/ESI-QTOF-MS system in negative ion mode utilizing the 6530B Accurate-mass-QTOF-MS (Agilent Technologies, Inc., Santa Clara, CA, USA) mass spectrometer with an ESI-Jet Stream ion source system. The Agilent 1260 chromatograph was equipped with a DAD detector, autosampler, binary gradient pump, and column oven. Gradients of solvents, water with 0.1% formic acid (solvent A) and acetonitrile with 0.1% formic acid (solvent B), were used as the mobile phases. The following gradient procedure was adopted: 0–7 min, 15–35% of B; 7–20 min, 35–40% B; 20–42 min 40–65% B, 42–43 min 65–95% B, 43–50 min 95% B; the post time was 10 min. Total time of analysis was 60 min, with a stable flow rate at 0.300 mL/min. For the stationary phase Phenomenex Luna Omega Polar C18 (Phenomenex, Torrance, CA, USA), 100 × 2.1 mm, dp 3 um was used. The injection volume for extracts was 10 μL. ESI-QTOF-MS analysis was performed according to the following parameters of the ion source: Dual spray jet stream ESI; positive and negative ion mode; gas (N2) flow rate: 12 L/min.; nebulizer pressure: 35 psig; vaporizer temp.: 300 °C; *m*/*z* range 100–1000 mass units, with acquisition Mode Auto MS/MS; collision-induced dissociation (CID): 10 and 30 eV with MS scan rate 1 spectrum per s, 2 spectra per cycle; skimmer: 65 V; fragmentor: 140 V; and octopole RF Peak: 750 V.

The identification was performed on the basis of MS/MS spectra. Identification was performed with use of open access MS-Dial 5.1.230719 software [36], and this was compared with literature data.

### 4.3. Phenolic Acid Content

The content of phenolic acids in the tested extracts was determined using a spectrophotometric method, based on the procedure described in the Polish Pharmacopoeia X [37]. Samples of approximately 1 g of *S. scardica* herb were prepared and extracted three times (30 min each) with methanol (Chempur, Pol-Aura, Warsaw, Poland) as the solvent. The combined extracts were then filtered, the solvent was evaporated, and 20 mL of hot distilled water was added. The samples were subsequently stored at 4 °C for 12 h.

After incubation, the solutions were filtered into volumetric flasks and diluted with distilled water to a final volume of 100 mL. From the resulting extracts, 1 mL was taken and mixed sequentially with 1 mL of distilled water, 1 mL of 0.5 M hydrochloric acid, and 1 mL of Arnow’s reagent (Chempur, Piekary Śląskie, Poland). After 6 min, 1 mL of 1 M sodium hydroxide solution (Chempur, Piekary Śląskie, Poland) and 5 mL of distilled water were added. The final mixture was transferred to a cuvette, and the absorbance was measured at 490 nm using a Hitachi U-2900 UV-Vis model spectrophotometer, (Hitachi High-Tech Corporation, Ibaraki, Japan) against a control sample (reagent mixture without extract).

The phenolic acid content was expressed as milligrams of caffeic acid equivalent per gram of extract (mg/g).

### 4.4. Flavonoid Content

The flavonoid content was determined spectrophotometrically according to the methodology described in the Polish Pharmacopoeia V [38], using solutions obtained after extraction of the dried plant material. The extraction was performed with a solvent mixture consisting of acetone (Chempur, Piekary Śląskie, Poland), hydrochloric acid (250 g·L^−1^; Chempur, Piekary Śląskie, Poland), and methenamine (5 g/L; Merck, Poznań, Poland). For the analysis, 1 g of crushed *S. scardica* herb was used and extracted three times.

Absorbance was measured at 425 nm using a Hitachi U-2900 UV-Vis model spectrophotometer, (Hitachi High-Tech Corporation, Ibaraki, Japan) against an appropriate reference solution. The results were expressed as total flavonoids (mg·g^−1^ dry weight), calculated as quercetin equivalents.

### 4.5. Biological Activity of the Sideritis Herba

#### 4.5.1. Polyphenols Content

The total polyphenol content in *S. scardica* herb was determined using the Folin–Ciocalteu method, with minor modifications, according to the procedures described by Singleton and Rossi [39] and Turkmen et al. [40]. One gram of crushed herb was weighed and placed in a flat-bottomed flask, followed by the addition of 50 mL of methanol (Chempur, Piekary Śląskie, Poland). The prepared plant material was extracted under a reflux condenser in a water bath for 30 min. The extraction was repeated three times, each time using 50 mL of methanol.

To determine phenolic content, 0.1 mL of the methanol extract was mixed with 6 mL of distilled water and 0.5 mL of Folin–Ciocalteu reagent (Chempur, Piekary Śląskie, Poland). After thorough mixing, the solution was left to stand for 3 min. Then, 1.5 mL of saturated sodium carbonate (Chempur, Piekary Śląskie, Poland) solution and 1.9 mL of distilled water were added. The mixture was incubated at 40 °C for 30 min in a thermostat. Absorbance was measured at 765 nm against a reagent blank (without extract) using a Hitachi U-2900 UV-Vis model spectrophotometer, (Hitachi High-Tech Corporation, Ibaraki, Japan).

The results were calculated based on a standard calibration curve prepared using gallic acid and expressed as milligrams of phenolic compounds per gram of dry weight (mg GAE/g DW).

#### 4.5.2. Assessment of Antioxidant Activity by the DPPH Radical Scavenging Assay

The antioxidant activity of methanol extracts from dried Sideritis herb was determined using the DPPH method, which involves a colorimetric measurement of the degree of reduction of DPPH (2,2-diphenyl-1-picrylhydrazyl; Merck, Poznań, Poland) free radicals. The absorbance was measured at 517 nm using a Hitachi U-2900 UV-Vis model spectrophotometer, (Hitachi High-Tech Corporation, Ibaraki, Japan) and methanol as a reference solution. Each analysis was performed in triplicate. A 1% solution of ascorbic acid ((vitamin C; Sigma-Aldrich, St. Louis, MO, USA)) in methanol was used as a control. To prepare the extract, approximately 2 g of the sample was poured with 50 mL of methanol and then left for 24 h in a dark place. After this time, the solution was filtered and used for further analyses. A blank sample was prepared by replacing the extract with water. The analytical procedure was performed according to the method described by Yen and Chen [41]. The results were expressed as % inhibition of DPPH radicals according to the formula proposed by Rossi et al. [42]:

X% = 100 − (At/Ar × 100)

At—absorbance of the test sample,

Ar—absorbance of blank test.

#### 4.5.3. Assessment of Antioxidant Activity by the FRAP (Ferric Reducing Antioxidant Power) Assay

The antioxidant activity of the methanol extracts of the tested plant material was determined using the FRAP (Ferric Reducing Antioxidant Power) method, following the procedure described by Thaipong et al. [43] and Mulugeta et al. [44], with slight modifications. For the analysis, 2 g of Sideritis herb was weighed and extracted with 50 mL of methanol (Chempur, Pol-Aura, Warsaw, Poland). The extract was left to stand for 24 h. After this period, the solution was filtered, and 100 μL of the extract was taken for further analysis.

The FRAP reagent was freshly prepared before use by mixing acetate buffer (0.3 M), TPTZ solution, and iron(III) chloride hexahydrate solution (20 mM) in a volume ratio of 10:1:1 (all reagents from Sigma-Aldrich, St. Louis, MO, USA or Chempur, Pol-Aura, Poland). For the assay, 3 mL of the freshly prepared FRAP reagent and 100 μL of the sample extract were combined in test tubes and incubated at 37 °C for 10 min. After incubation, absorbance was measured at 593 nm against a blank (FRAP reagent without extract) using a Hitachi U-2900 UV-Vis model spectrophotometer, (Hitachi High-Tech Corporation, Ibaraki, Japan).

The antioxidant capacity was calculated based on a standard curve prepared using Trolox, and the results were expressed as Trolox equivalents (mg Tr/g dry weight).

#### 4.5.4. The Antimicrobial Activity of the Sideritis Herb Extracts

The antimicrobial activity of the *Sideritis scardica* Griseb. extracts was evaluated using the broth microdilution method, in accordance with the guidelines of the European Committee on Antimicrobial Susceptibility Testing (EUCAST) [45], as previously described [46]. For antimicrobial testing, the methanol extract was first concentrated by evaporating methanol under reduced pressure. The resulting thick extract was then dissolved in dimethyl sulfoxide (DMSO), which was used as the solvent for preparing test solutions.

Serial dilutions of the *S. scardica* extracts were prepared to achieve final concentrations of 16, 8, 4, 2, 1, 0.5, 0.25, 0.125, 0.06, and 0.03 mg/mL. This method was employed to determine the minimum inhibitory concentration (MIC), minimum bactericidal concentration (MBC), or minimum fungicidal concentration (MFC) of each essential oil against twenty-three microbial strains under in vitro conditions.

The antimicrobial efficacy of the *S. scardica* extracts was assessed against reference strains obtained from the American Type Culture Collection (ATCC), comprising twelve Gram-positive bacteria strains (*Staphylococcus aureus* ATCC 29213, ATCC 6538P, ATCC 25923—methicillin-sensitive; *Staphylococcus aureus* ATCC 43300, ATCC BAA1707—methicillin-resistant; *Staphylococcus epidermidis* ATCC 12228; *Enterococcus faecalis* ATCC 29212, ATCC 51299; *Enterococcus faecium* ATCC 19434; *Micrococcus luteus* ATCC 10240; *Bacillus subtilis* ATCC 6633; *Bacillus cereus* ATCC 10876); eleven Gram-negative bacteria strains (*Salmonella enteritidis* ATCC13076; *Salmonella* Typhimurium ATCC 14028; *Proteus mirabilis* ATCC 12453; *Bordetella bronchiseptica* ATCC 4617; *Escherichia coli* ATCC 25922 and ATCC 35218; *Klebsiella pneumoniae* ATCC 13883 and ATCC BAA2146; *Enterobacter aerogenes* ATCC 13048; *Pseudomonas aeruginosa* ATCC 27853; *Acinetobacter baumanii* ATCC 19606); and twelve yeast strains (*Candida albicans* ATCC 2091, ATCC 10231, 14053; *Candida auris* CDC B11903; *Candida glabrata* ATCC 90030, ATCC 15126; *Candida parapsilosis* ATCC 22019; *Candida krusei* ATCC 14243; *Candida lusitaniae* ATCC 34449; *Candida tropicalis* ATCC 1369; *Geotrichum candidum* ATCC 34614; *Candida glabrata* ATCC 66032).

All assays were performed in triplicate. Standard antimicrobial agents were used as positive controls: fluconazole (0.06–16 µg/mL) for yeasts, ciprofloxacin (0.015–16 µg/mL) for Gram-negative bacteria, and vancomycin (0.06–16 µg/mL) for Gram-positive bacteria. The MIC values obtained for these reference compounds were 1 µg/mL fluconazole for *C. albicans* ATCC 10231, 1 µg/mL vancomycin for *S. aureus* ATCC 29213, and 0.015 µg/mL ciprofloxacin for *E. coli* ATCC 25922.

### 4.6. Statistical Analysis

The obtained results are presented as the means and were statistically analyzed by ANOVA, and the averages were compared using Tukey′s HSD test at the probability level α = 0.05. Statistical analyses were calculated with Statistica 13.3 PL software (StatSof Inc., Tulsa, OK, USA).

## 5. Conclusions

The genus Sideritis includes morphologically and chemically diverse herbaceous plants and subshrubs, found in various habitats and at different altitudes. Due to their richness in biologically active compounds such as terpenes, phenols, and essential oils, these plants exhibit a broad spectrum of pharmacological properties, including anti-inflammatory, antimicrobial, and antioxidant effects. The polyphenols they contain—particularly verbascoside and chlorogenic acid—are primarily responsible for their strong antioxidant activity. The analyzed extracts of *S. scardica* showed variations in chemical composition depending on geographical origin, with verbascoside identified as the dominant compound in all samples. Chlorogenic acid was also detected and, alongside verbascoside, may contribute to the antimicrobial and antifungal properties, especially against Gram-positive pathogens and Candida spp. Studies indicate that phenotypic and environmental variability influence the chemical composition of the raw material and, consequently, its biological activity—an observation confirmed by the present research. The results highlight the therapeutic potential of *S. scardica* extracts, particularly those originating from Türkiye (Sh2), and emphasize the need for further investigation into the identification of active compounds and their synergistic effects with conventional drugs.

## Figures and Tables

**Table 1:** Results of ESI-QTOF-MS analysis of methanolic extracts from *S. scardica* herb.

No	Tentative Assignment	Retention Time [min]	Formula	[M–H]^−^	MS/MS Fragments [*m*/*z*]
1	Isoacetoside	1.486	C_29_H_36_O_15_	623.1955	461.1628; 161.0218; 113.0222
2	Quinic acid	1.536	C_7_H_12_O_6_	191.0526	171.0249; 127.0377; 109.0268
3	Malic acid	1.636	C_4_H_6_O_5_	133.0114	115.0015
4	Citric acid	2.186	C_6_H_8_O_7_	191.0197	110.9879
5	Chlorogenic acid	2.719	C_16_H_18_O_9_	353.0672	191.0434
6	Vicenin	3.286	C_27_H_30_O_15_	593.1531	473.1088; 503.1198; 383.0764; 353.0657; 191.0541
7	5-O-Caffeoylquinic acid methyl ester	5.702	C_17_H_20_O_9_	367.1027	179.0358; 161.0249; 135.0453
8	Coumaric acid	5.936	C_9_H_8_O_3_	163.0412	119.0491
9	Verbascoside	8.486	C_29_H_36_O_15_	623.1874	461.1574; 161.0188; 113.0203
10	Forsythoside B	10.620	C_29_H_36_O_15_	755.2485	623.2054; 593.2137; 461.1716; 161.0243
11	Plantainoside C	10.986	C_30_H_38_O_15_	637.2201	461.1701; 315.1092; 193.0497; 175.0393; 161.0273; 113.0234
12	Alyssonoside	11.403	C_35_H_46_O_19_	769.2624	623.2033; 593.2122; 461.1693; 193.0501; 175.0393; 161.0243
13	Kaempferol dihexoside	11.453	C_27_H_30_O_16_	609.1496	429.0850; 285.0410
14	Apigetrin	12.653	C_21_H_20_O_10_	431.1033	268.0397
15	Rhamnocitrin hexoside	15.987	C_22_H_22_O_11_	623.1626	299.0551
16	Apigenin	20.288	C_15_H_10_O_5_	269.0473	151.0037; 117.0344
17	Patuletin 3-rhamnoside-7-(3‴,4‴-diacetylrhamnoside)	22.821	C_36_H_32_O_16_	707.1943	299.0587; 101.0243
18	Luteolin dimethyl ether	23.888	C_17_H_14_O_6_	313.0701	298.0546; 283.0274
19	Eupatorin	25.154	C_18_H_16_O_7_	343.0808	313.0386; 298.0142; 270.0191; 193.0140; 186.0331
20	Velutin	27.888	C_17_H_14_O_6_	313.0739	298.0495; 283.0253

**Table 2:** The content of flavonoids and phenolic acids in *S. scardica* herb extracts (Sh1—herb from Bulgaria; Sh2—herb from Türkiye; Sh3—herb from Macedonia; Sh4—herb from Albania).

Type of Material	Flavonoids		
	Mean (mg/g)	Standard Deviation	Standard Error
Sh 1	3.96	0.116	0.067
Sh 2	2.50	0.378	0.218
Sh 3	2.50	0.462	0.266
Sh 4	6.18	0.796	0.460
Mean	3.79	0.116	0.067
**Type of Material**	**Phenolic Acids**		
	**Mean (mg/g)**	**Standard Deviation**	**Standard Error**
Sh 1	11.4	0.068	0.039
Sh 2	19.1	0.413	0.238
Sh 3	16.4	0.321	0.186
Sh 4	21.8	0.246	0.142
Mean	17.2	0.068	0.039

**Table 3:** Description and characteristics of plant material.

Material Designation	Name	Origin	Producer
Sh 1	*S. scardica* herb	Bulgaria	PPH Astron; Stary Henryków, Poland
Sh 2	*S. scardica* herb	Türkiye	Bakra Natura; Gdańsk, Poland
Sh 3	*S. scardica* herb	Macedonia	Bakra Natura; Gdańsk, Poland
Sh 4	*S. scardica* herb	Albania	Farm Vit; Piotrków Trybunalski, Poland

## Data Availability

The original contributions presented in the study are included in the article, further inquiries can be directed to the corresponding author.

## Abbreviations

The following abbreviations are used in this manuscript:

DPPH	2,2-diphenyl-1-picrylhydrazyl
FRAP	Ferric Reducing Antioxidant Power
Sh 1	*S. scardica* herb from Bulgaria
Sh 2	*S. scardica* herb from Türkiye
Sh 3	*S. scardica* herb from Macedonia
Sh 4	*S. scardica* herb from Albania
